# Inflammation and macrophage polarization are associated with Modic change type in lumbar radiculopathy

**DOI:** 10.1016/j.bas.2025.104249

**Published:** 2025-03-26

**Authors:** Wensen Li, Niek Djuric, Christiaan Mink, Carmen L.A. Vleggeert-Lankamp

**Affiliations:** a Neurosurgical Center Holland, Leiden University Medical Center & Haaglanden Medical Center & Haga Teaching Hospital, Hoofddorp, Haarlem, the Netherlands; b Spaarne Gasthuis, Hoofddorp, Haarlem, the Netherlands

**Keywords:** Intervertebral disc herniation, Lumbar, Inflammation, Macrophage, M1 and M2, Modic changes

## Abstract

**Introduction:**

Lumbar disc herniation (LDH) typically manifests as sciatica, attributed to nerve root mechanical compression and/or inflammation. Modic changes (MC), classified as type I or type II, are deemed to represent endplate vascular insufficiency and are hypothesized to create an inflammatory environment. Macrophages infiltrating disc tissue can be polarized into pro-inflammatory M1 or anti-inflammatory M2 phenotypes.

**Research question:**

This study aims to investigate the interplay among inflammatory cells, including M1 and M2 macrophages, Modic Changes, and hernia size and type in patients suffering from sciatica due to a LDH.

**Material and methods:**

This prospective cohort study selected patients undergoing microdiscectomy for LDH. Macrophage infiltration (CD68, CD192, CD163), MC classification on MRI, and hernia parameters were analyzed.

**Results:**

132 out of 187 patients demonstrated macrophages in the lumbar disc tissue. Most samples demonstrated severe inflammation (50 %), and most macrophages were of the M1 phenotype (48 %). MC were present in 45 % of patients, and only 19 % of these demonstrated MC type I. MC type I were highly associated with both severe (p = 0.016) and M1 macrophage-dominant inflammation (p = 0.048). Larger and non-contained herniations associated with increased inflammation (p = 0.029/p = 0.002), while larger herniations associated with the presence of MC type II (p = 0.027).

**Discussion and conclusions:**

This study elucidates a close association of MC type I and M1 macrophage. MC type II were observed more often in patients with larger HNPs. This is indicative for MC typing as an important factor in prediction modelling and it suggests the potential for personalized treatment strategies.

## Highlights

• MC was not associated with inflammation or macrophage dominance, but MC type I was associated with severe inflammation.
• MC type I was associated with M1 dominance, whereas MC type II was not significantly associated with inflammation or macrophage type.
• Large HNPs were associated with severe inflammation, but HNP size was not associated with the macrophage dominance type.
• Both MC types were associated with non-contained disc herniation; only Modic type II was associated with larger HNP.

## 1 Introduction

Lumbar disc herniation (LDH) is a common condition characterized by the displacement of nucleus pulposus material beyond the disc space, often resulting in compression of adjacent neural structures and the onset of a lumbar radiculopathy syndrome (LRS) (Rajasekaran et al., 2013). An important aspect of the pathophysiology of LRS revolves around the inflammatory response induced by herniated disc material within the epidural space, in particular the infiltration of macrophages (Djuric et al., 2020a). It is hypothesized that herniated nucleus pulposus (HNP) material triggers a foreign body reaction, which induces macrophage infiltration into the affected area (Djuric et al., 2019). While these macrophages are thought to participate in the resorption of herniated disc material, they also contribute to the induction of an inflammatory cascade, potentially exacerbating pain symptoms (Koroth et al., 2023). The role of macrophages in LDH-induced inflammation is still controversial, with different opinions based on the different phenotypic characteristics of macrophages in the disc environment (Li et al., 2023).

The current literature distinguishes between two main phenotypes of macrophages: M1 and M2. M1 macrophages, stimulated by factors such as lipopolysaccharide (LPS), interferon-gamma (IFN-γ) and tumor necrosis factor (TNF), exhibit a pro-inflammatory profile characterized by the secretion of cytokines such as IL-1β, IL-6, IL-12 and TNF-α. These pro-inflammatory mediators have been implicated in nociceptive sensitization and the exacerbation of pain symptoms associated with LDH (Martinez et al., 2014; Arango Duque and Descoteaux, 2014; Dube et al., 2017; Pedersen et al., 2015). Conversely, M2 macrophages, induced by factors such as IL-4, IL-13 and glucocorticoids, have an anti-inflammatory profile and secrete cytokines such as IL-10 and transforming growth factor-beta (TGF-β), which promote tissue repair and remodeling and may alleviate pain symptoms by resorbing herniated disc material (Martinez et al., 2014; Zhang et al., 2020; Shapouri-Moghaddam et al., 2018). Despite numerous studies, the exact role of M1 and M2 macrophages in LDH-related inflammation and pain remains unclear. A review by Djuric et al. (2020b) identified that M1-related pro-inflammatory cytokines, such as TNF-α, TNFR1, IL-6, IL-8, and IFN-γ, were associated with higher pain scores, while M2-related anti-inflammatory cytokines, including IL-4 and IL-10, were linked to lower pain scores. This finding aligns with the hypotheses mentioned above.

It has been shown that the presence of Modic Changes (MC) on Magnetic Resonance Imaging (MRI), also known as Vertebral Endplate Signal Changes (VESC), is not only representing vascular insufficiency of the endplate, but also appears to be associated with inflammation (Urquhart et al., 2015; Karppinen et al., 2021). There are indications that MC type I is associated with an autoimmune response to a pro-inflammatory environment (Dudli et al., 2016), and even can be caused by infection (Crockett et al., 2017). MC type I patients with Cutibacterium acnes (formerly named Propionibacterium acnes) infections have demonstrated an innate immune cell profile and upregulation of pro-inflammatory cytokines (Heggli et al., 2024).

In a pilot study we evaluated the M1 and M2 characterization in disc material in the presence/absence of Modic Changes, and confirmed that higher levels of CD68^+^ (macrophage staining) in symptomatic lumbar disc herniations are associated with the presence of MC. Moreover, we demonstrated a relatively overexpression of M2 markers if MC were absent (Djuric et al., 2019). We hypothesize that Modic changes are associated with a dominance of M1 macrophage infiltration, although this could not be demonstrated using the limited data in the pilot study. As MC were demonstrated to be closely associated with inflammatory processes and pain symptoms in the lumbar spine, and macrophages play a crucial role in regulating the inflammatory milieu, a deeper investigation of the intrinsic link between different MC types and M1/M2 macrophages is of great significance.

Additionally, literature has provided evidence that biomarkers of M1 and M2 macrophages are significantly increased in regions of the nucleus pulposus (NP), annulus fibrosus (AF) and endplate (EP) that exhibit structural irregularities and defects (Nakazawa et al., 2018). In an earlier study, we also demonstrated that the degree of macrophage infiltration was higher in non-contained herniated discs (extrusion) in comparison to contained disc herniations (Djuric et al., 2020a).

Thus, the aim of this study was to further explore the association between different macrophage types (M1 and M2) and Modic changes (MC type I and MC type II), as well as their association with containment of the herniated disc, to deepen our understanding of pain mechanisms of LRS due to a herniated disc.

## 2 Methods

### 2.1 Study design

A multicenter, prospective observational cohort study was conducted among patients with lumbosacral radiculopathy due to a herniated disc, confirmed by MRI evaluation. The protocol was approved in all four participating centers by the Medical Ethics Committee of the Leiden University Medical Center, and subsequently by the Board of Directors of the Alrijne Hospital Leiderdorp/Leiden, Spaarne Gasthuis Haarlem/Hoofddorp and the HAGA hospital the Hague (P18.211). Written informed consent was obtained from all patients. Details of the protocol have been published previously (Djuric et al., 2021). This trial was registered with the Netherlands Trial Register (no. NL8464, www.trialregister.nl).

### 2.2 Participants

Patients were eligible if they fulfilled the inclusion criteria: age between 18 and 75 years old, presence of a radiologically (MRI) proven herniated disc consistent with clinically relevant lumbar radiculopathy, patient to be eligible and scheduled for lumbar discectomy, and presence of disabling clinical symptoms for more than 8 weeks (Djuric et al., 2021). Exclusion criteria were previous lumbar spinal surgery, leg paresis (MRC <4), history of spinal inflammatory disease, instability requiring instrumented spondylodesis surgery, active infection at the time of surgery, and use of antibiotics in the six months preceding surgery. Other exclusion criteria were short-term planned migration, no or limited understanding of the Dutch language, and pregnancy.

### 2.3 Intervention

Unilateral transflaval microdiscectomy was performed in all patients. The level of the incision was determined using fluoroscopy. After a small midline incision, the muscle was detached from the spinous processes. A small horizontal hemilaminectomy at the side of the hernia was performed and the flaval ligament was reduced. Removal of the herniated disc material was performed and continued until the nerve root was completely decompressed. The resected disc material was collected and stored in 4 % formaldehyde and within hours transported to the LUMC pathology laboratory.

### 2.4 Immunohistochemistry

The harvested disc tissues were fixed in 4 % formaldehyde solution for 3–7 days. Tissue was subsequently embedded in paraffin blocks and 5-μm thick slices were taken from the middle of the block for haematoxylin and eosin staining, which was performed according to the Leica ST 5020-mulitstainer standard protocol. Samples were evaluated under the microscope for presence of inflammatory cells. If tissue from one sample exceeded the capacity of 1 paraffin block, multiple blocks were produced and a slide of each block was evaluated.

**Fig. 1:**
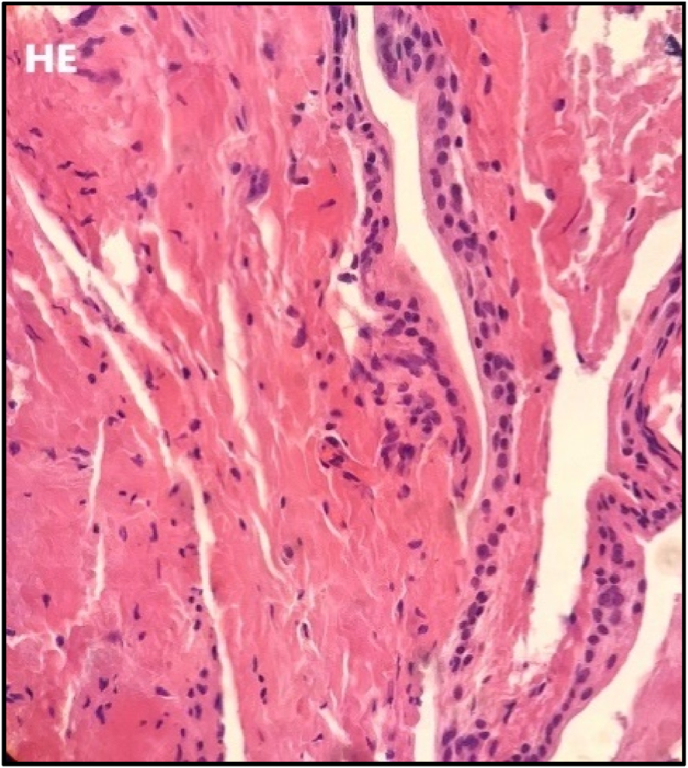
HE staining screened samples for the presence of inflammatory cells.

**Fig. 2:**
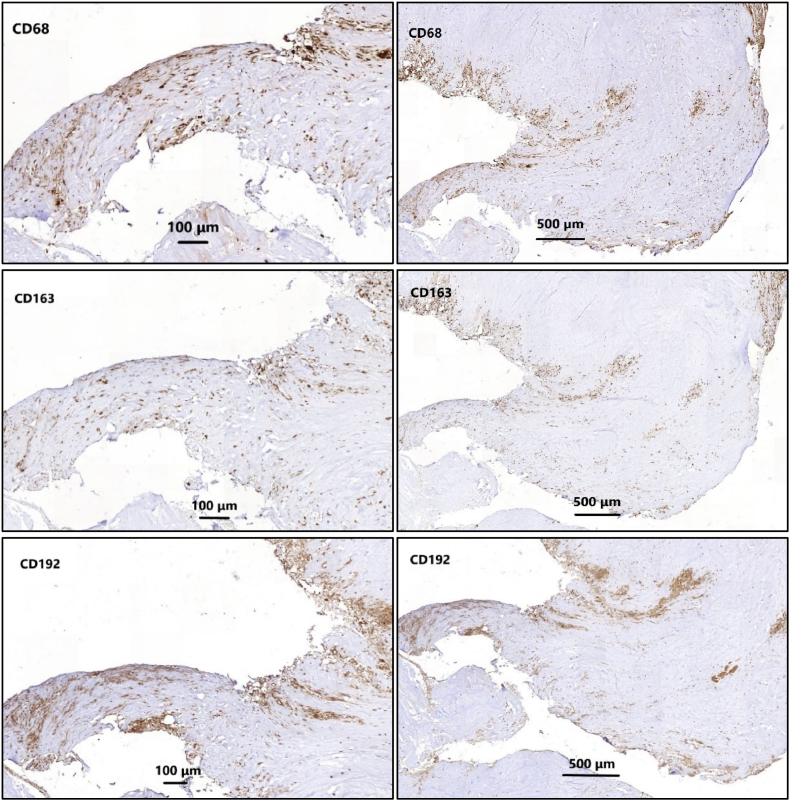
Image scan of serial sections of the same tissue wax block stained with antibodies against CD68, CD163 and CD192, respectively. ImageJ was applied for algorithmic counting of brown-positive macrophages.

In case of positive HE stained cells, additional slices were prepared for immunohistochemistry staining. For the staining procedure, three serially cut 5-μm paraffin slices were rinsed in ethanol and methanol solutions and prepared for the expression of CD68 (to visualize macrophages; DAKO Denmark), CD192 (to visualize macrophage type 1: M1; Thermo Fisher Scientific, Netherlands), and CD163 (to visualize macrophage type 2, M2; Abcam, Netherlands), respectively. Immunohistochemistry was performed using a three-step indirect method. Antibodies CD68 were cooked in Citrate pH 6.0 buffer, CD 192 and CD 163 were cooked in EDTA pH 8.5 buffer as a pre-treatment. Subsequently, an avidin-biotin complex technique was performed with the Vectastain ABC-Elite Kit (Vector Lab. USA) and the appropriate biotinylated antibodies. Visualization of the peroxidase reaction was done with DAB solution (Sigma). Samples were counterstained with Harris haematoxylin. All samples were accompanied by a positive control, which was atherosclerosis tissue for all macrophage markers (Djuric et al., 2021). (Fig. 1) For evaluation all samples were photographed using Philips ultra-fast scanner (see Fig. 2).

### 2.5 Macrophage counting

Cell counts were performed using ImageJ and evaluation was executed by two independent researchers (ND & WL). Inter-observer correlation coefficients were calculated for each staining separately. For each antibody 50 pictures were evaluated by hand: cells were analyzed based on morphological features and only macrophages were photographed and evaluated. Subsequently an automated cell count algorithm in ImageJ was matched on the average count of the two observers with a correlation coefficient of >0.8, which was regarded as a strong correlation. This algorithm and parameter settings were used for evaluation on all images. Positive macrophage/lymphocyte counts were divided by the surface of the evaluated herniated disc material in cm2.

First samples were evaluated for the presence of HE positive cells to determine whether inflammatory cells were present. Thereafter the samples were evaluated based on the presence of CD68 staining and categorized: no inflammation (0 macrophages; including the samples that did stain negative with the HE staining), mild (1–10 macrophages per cm2, moderate (10–100 macrophages per cm2) and severe (>100 macrophages per cm2) inflammation.

Subsequently, M1 and M2 dominance was evaluated in the samples containing CD68 positive cells: it was only determined if at least 10 positive CD68 cells/cm^2^ were present. For relative M1/M2 dominance, M1 and M2 markers were expressed as percentages (ranging from 0 % to 100 %) of the number of CD68 positive cells. M1 dominance was defined when the percentage of M1 cell counts was more than 60 %. If the percentage of M1 was between 40 % and 60 %, it is defined as M1 equal to M2. M2 dominance was defined if the percentage of M1 cell count was less than 40 %.

### 2.6 MRI

For the evaluation of Modic Changes, a 3T MRI was used. Both sagittal T1-and T2-weighted images of the lumbar spine were obtained. Image evaluation of MC was according to the criteria of Modic et al., 1988a, 1988b. Image evaluation was done by two independent researchers (ND and WL) and one neurosurgeon (CVL) in a blinded manner. Inter agreement analysis was performed and kappa values were calculated. Upon disagreement, a final judgement was made in open discussion.

### 2.7 Modic change evaluation

MRI scans were performed at baseline by a 1.5 T scanner, and both sagittal T1-and T2-weighted images of the lumbar spine were evaluated. MRIs were evaluated by 2 investigators (WL & ND) and 1 neurosurgeon (CVL). All three were blinded to histological data and clinical information. The readers were not involved in the selection or treatment of the patients included. Modic changes were assessed and classified as present or absent, and scored as Type I, Type II, or Type III, according to the criteria of Modic et al.

### 2.8 Size of hernia evaluation

Using adjusted evaluation criteria mentioned in previous research, the size of herniated nucleus pulposus (HNP) as quantified by MRI was categorized as follows: bulging, small (<25 % of vertebral canal), average (25–50 %), large (50–75 %), and stenosis (>75 % of spinal canal) (Suthar et al., 2015; Hao et al., 2017; Lee et al., 2017).

The shape of the hernia was also evaluated on the sagittal view with reference to the recommendations for nomenclature and classification of lumbar disc pathology (Fardon et al., 2001): if the herniated material remained at the level of the disc, this was classified as ‘protrusion’. If the herniated material reached beyond the level of the disc, it was classified as ‘extrusion’. Depending on whether the nucleus pulposus breaks through the annulus fibrosus, the hernia was dichotomously classified as ‘contained disc’ (bulging and protrusion), while extrusion and sequestration were classified as ‘non-contained disc’.

### 2.9 Statistics

For the evaluation of the association of MRI and histological data with clinical data, Monte Carlo simulations were performed in the statistical software R to calculate the appropriate sample size, requiring 160 patients (Djuric et al., 2021). The current study evaluating MRI and histological data is an exploratory study.

Descriptive statistics for all baseline data were expressed as mean ± SD or as N. For comparing numerical data, Kruskal-Wallis tests were used. Associations between categorical variables, including the associations between the degree of inflammation, macrophage dominance and Modic changes (MC) were assessed using Fisher's exact tests. Inflammation was not only associated with MC using the 4 indicated categories, but also after dichotomizing the degree of inflammation into ‘minimal’ (<10 macrophages per cm^2^) and ‘considerable’ (>10 macrophages per cm^2^).

At last, due to the smaller sample size per group: Fisher's exact test was used to explore the association between macrophage polarization, MC and HNP size and containment. All statistical analyses were performed using IBM SPSS software version 29.0.0.0. P-values of <0.05 were regarded as significant.

## 3 Results

### 3.1 Characteristics of the study population

Between June 2019 and October 2023, a total of 237 consecutive patients scheduled for lumbar disc surgery for LRS were assessed eligible for inclusion. After screening according to the exclusion criteria, a total of 187 patients, with a mean age of 47 years enrolled in the study. There were no data excluded due to quality control concerns. 56 % of patients was female, and the mean BMI was 26.5. Disc herniation was most commonly present at the L5-S1 level (49 %), followed by the L4-5 level (44 %) (Table 1).

**Table 1:** Overview characteristics of the three types of Modic changes. (N = 187).

	No MC (N = 102)	MC I (N = 16)	MC II (N = 69)	P value
Age (y)	45.6 ± 13.2	51.6 ± 10.3	47.6 ± 11.4	0.416^a^
Male gender	43 %	50 %	43 %	0.903
BMI	26.8 ± 5.0	25.4 ± 3.4	26.2 ± 4.1	0.709^a^
HNP level				0.479
L2-L3 (N = 3)	3	0	0	
L3-L4 (N = 9)	5	1	3	
L4-L5 (N = 83)	46	4	33	
L5-S1 (N = 92)	48	11	33	

Fisher's exact test was performed to calculate other P values.

a P-value calculated using Kruskal-Wallis Test.

### 3.2 Histology and immunohistological data analysis

Evaluation of the HE staining results revealed that inflammatory cells were present in 140 (75 %) of 187 patients (kappa value of 0.94). CD68 staining to identify macrophages showed the following distribution: 55 did not stain positive for macrophages (negative in HE staining and negative in CD68 staining) (29 %), 8 (4 %) patients were classified as having mild inflammation, 31 (17 %) as having moderate inflammation, and 93 (50 %) as having severe inflammation.

The subsequent evaluation of the CD163 and CD192 characterization of macrophages revealed that two of the samples that stained negative for CD68 antibodies did stain positive for CD163 and CD192, coming to a total of 134 patients out of 187 displaying macrophages. The M1 phenotype predominated in the majority 89 of 134 samples, type M2 predominated in 17 patients, and M1 equaled M2 in 28 patients (Table 2).

**Table 2:** Associations between MC and histological results.

Fisher's exact test	Category	N	No MC	MC I	MC II	P value
Inflammation severity (CD68)						0.160
	**No staining (0)**	55	29	1	25	
	**Mild (1**–**10)**	8	4	0	4	
	**Moderate (10**–**100)**	31	20	3	8	
	**Severe (>100)**	93	49	12	32	
Inflammation severity (CD68) dichotomized						0.016∗
						
	**Minimal (0**–**10)**	63	33	1	29	
	**Considerable (>10)**	124	69	15	40	
Macrophages Dominance						0.235
(CD192)	**M1 dominant**	89	49	12	28	
(CD192 + CD163)	**M1 equals M2**	28	16	2	10	
(CD163)	**M2 dominant**	17	10	1	6	

∗ P-value <0.05.

### 3.3 Modic changes

MC at the level of the lumbar disc herniation were present in 85 of 187 (45 %) patients. MC type II was identified in 69 patients and MC type I was present in 16 patients. No type III Modic changes were observed (Table 1). The interobserver agreement was 82 %, with a kappa value of 0.72.

### 3.4 Size and containment of herniated discs

Small (70 patients) and average (62 patients) disc herniation sizes were most common, while bulging disc (16 patients), large (21 patients) and very large stenosing disc herniations (18 patients) were less common. Dichotomization of disc size, considering bulging and small herniations as ‘small HNP’, demonstrated that 46 % of patients demonstrated a ‘small HNP’, and 54 % a ‘large HNP’. The results showed that 36 % of the patients had a contained HNP; the remainder had a non-contained HNP.

### 3.5 Association between inflammation parameters and MC

The presence of MC was neither associated with the severity of inflammation (CD68 staining, p = 0.160), nor with the macrophage type predominance (CD192 or CD163 staining, p = 0.235) (Table 2, Table 3). However, after dichotomizing the degree of inflammation into ‘minimal’ (<10 macrophages per cm2) and ‘considerable’ (>10 macrophages per cm2) and dividing MC-positive patients into MC types I and II, it was observed that there was an association between severity of inflammation and the presence of MC type I (p = 0.016) (Table 2). Further exploration considering also the macrophage polarization revealed that if MC type I was present, this was associated with the presence of M1 macrophages (p = 0.048) (Table 3). By contrast, MC type II showed a similar degree of inflammation and similar type of macrophage infiltration as patients without MC.

**Table 3:** Associations between dichotomized MC and inflammation type.

Fisher's exact test	N	No MC (N = 102)	MC I (N = 16)	MC II (N = 69)	P value
					
M1					0.048∗
No dominant	98	53	4	41	
Dominant	89	49	12	28	
M2					1.000
No dominant	170	92	15	63	
Dominant	17	10	1	6	

∗ P-value <0.05.

### 3.6 Association between inflammation parameters and HNP size/containment

Inflammation was more severe in the large HNP group (p = 0.029), but the size of the HNP was not associated with macrophage dominance (p = 0.895) (Table 4).

**Table 4:** Associations between HNP size and histological results.

Fisher's exact test	Category	N	HNP Small (N = 86)	HNP Large (N = 101)	P value
Inflammation severity (CD68)					0.003∗
	**No staining (0)**	55	33	22	
	**Mild (1**–**10)**	8	3	5	
	**Moderate (10**–**100)**	31	19	12	
	**Severe (>100)**	93	31	62	
Inflammation severity (CD68) dichotomized					0.029∗
	**Minimal (0**–**10)**	63	36	27	
	**Considerable (>10)**	124	50	74	
Macrophages Dominance					0.895
(CD192)	**M1 dominant**	89	36	53	
(CD192 + CD163)	**M1 equals M2**	28	10	18	
(CD163)	**M2 dominant**	17	7	10	

HNP Small = Bulging + Small <25 %; HNP Large = Average 25–50 % + Large 50–75 % + Stenosis >75 %.

∗ P-value <0.05.

The small size HNPs were usually contained, and the large ones were usually non-contained (Table 5). It was likewise demonstrated that non-contained disc herniations were associated with more severe inflammation (p = 0.002), but no association with macrophage dominance was established (p = 0.466).

**Table 5:** Associations between HNP type and histological results.

Fisher's exact test	Category	N	HNP Containment (N = 68)	HNP Non-containment (N = 119)	P value
HNP Size					<0.001∗∗∗
	**Small**	86	54	32	
	**Large**	101	14	87	
Inflammation severity (CD68)					<0.001∗∗∗
	**No staining (0)**	55	29	26	
	**Mild (1**–**10)**	8	4	4	
	**Moderate (10**–**100)**	31	15	16	
	**Severe (>100)**	93	20	73	
Inflammation severity (CD68) dichotomized					0.002∗∗
	**Minimal (0**–**10)**	63	33	30	
	**Considerable (>10)**	124	35	89	
Macrophages Dominance					0.466
(CD192)	**M1 dominant**	89	30	59	
(CD192 + CD163)	**M1 equals M2**	28	6	22	
(CD163)	**M2 dominant**	17	5	12	

HNP Containment = Bulging + Protrusion; HNP Non-containment = Extrusion + Sequestration.

∗∗ P-value <0.01; ∗∗∗ P-value <0.001.

### 3.7 Association between modic changes and HNP size

In dichotomizing the HNP size data into small and large, it was displayed that larger size HNP was associated with the presence of Modic type II changes (p = 0.027; Table 6) and not with the presence of MC type I. Both type I and type II MC were associated with non-contained HNP (p = 0.010), in contrast to the contained HNPs that were likely to be not accompanied by MC.

**Table 6:** Associations between MC and HNP.

Fisher's exact test	N	No MC (N = 102)	MC I (N = 16)	MC II (N = 69)	P value
HNP Size					0.052
Stenosis >75 %	18	11	2	5	
Large 50–75 %	21	8	3	10	
Average 25–50 %	62	28	3	31	
Small <25 %	70	43	6	21	
Bulging	16	12	2	2	
					
HNP Size dichotomized					0.027∗
Small	86	55	8	23	
Large	101	47	8	46	
					
HNP Type					0.010∗
Containment	68	47	4	17	
Non-containment	119	55	12	52	

∗ P-value <0.05.

## 4 Discussion

The current study results confirm the conclusion of a previous pilot study demonstrating an association between the presence of Modic changes and inflammation (Djuric et al., 2019). Modic changes are hypothesized to represent devascularization of the vertebral endplate, indicative for an environment that is receptive for inflammation. Currently, in a large group of patients in which a herniated disc was operated to treat a lumbar radicular syndrome we were additionally able to include the evaluation of associations with MC type I and II separately, and to classify the inflammatory cells into M1 and M2 type macrophages. It was demonstrated that only a minority of patients demonstrated MC type I, that this was associated with severe inflammation (p = 0.016), and that the vast majority of these inflammatory cells were of the M1 macrophage type.

Interestingly, our findings corroborate with those of Albert et al. (2013) (Albert et al., 2013), which showed that antibiotic treatment was more effective than placebo for patients with a history of lumbar disc hernia, still suffering from chronic low back pain and Modic type I changes. This suggests a plausible hypothesis: MC type I is thought to represent an acute or early degenerative process of the vertebral endplate where M1 macrophages (pro-inflammatory) are more prominent (Ohtori et al., 2006; Modic, 2007; Luoma et al., 2009). M1 macrophages are typically active in the early stages of tissue damage, promoting inflammation, and potentially linked to bacterial infection (Albert et al., 2008). It is hypothesized that patients may have a greater chance of benefiting from antibiotics or anti-inflammatory medicine in case inflammation plays a significant role. Our results indicate that the presence of MC type I is accompanied by the presence of M1 macrophages. In conclusion, this is the first time that findings on MC type and inflammation can be presented that are in line with hypotheses based on the remarkable findings of Albert.

In the current study, the Modic type II changes were more abundantly present than the Modic type I changes. However, Modic type II changes were not demonstrated to be coinciding with inflammation. Literature claims that MC type II represent a chronic or stable degenerative process with fibrosis and bone marrow fat replacement (Rahme et al., 2008). It is thus possible that these endplate changes remain after inflammation has subsided. This fits to the hypothesis that macrophages are absent in case type II MC are present, or that macrophages in the discs of those patients are of the M2 type (anti-inflammatory and recovery-promoting).

The AIM study, in which patients with chronic low back pain, previous disc herniation and Modic changes were randomized between antibiotic treatment and placebo, demonstrated a significant, though small and thus not clinically relevant, decrease in Roland Disability score in patients with MC type I, and not in patients with MC type II (Bråten et al., 2019). The authors conclude that their results do not confirm the results demonstrated by Albert et al. since the decrease in functionality was so limited. However, in the light of our findings, their results are in line with the results of Albert and with our hypothesis.

Furthermore, we observed that a larger, non-contained HNP is associated with more disc tissue inflammation. This is in agreement with our earlier findings demonstrating that lumbar disc extrusion decreased faster than bulging discs (Djuric et al., 2020a). This was hypothesized to be due to the active role of macrophages in the resorption process, and according to our hypothesis we would have expected for those macrophages to be of the M2 type. However, in the current study no such association could be demonstrated. This may be due to the limited number of samples studied. Future, larger, studies could explore this phenomenon more in depth.

Additionally, the correlation between the HNP size and MC was studied. Grading HNP size in five categories suggested an association between larger size and presence of MC and approached significance (Table 6, p = 0.052). However, the sample size was deemed too limited to reach significance. Therefore, the HNP size data were dichotomized, which revealed a significant association: If a hernia is large, MC are more likely to be of the type II than of the type I nature. With caution, this adds another hypothesis, namely that radicular pain in patients with type I MC, indicative of M1 macrophages, is more likely to be due to local irritation by cytokines and interleukins, and that the radicular pain in patients with type II MC is more likely to be caused by mechanical compression of the nerve by larger HNP tissue sizes.

These findings emphasize a more individualized treatment approach based on HNP characteristics (size, containment) and MC type. Patients with MC type I, in which inflammation plays a dominant role, and smaller, contained hernias may benefit more from conservative anti-inflammatory therapy, whereas patients with MC type II and larger, non-contained hernias may require a combination of decompressive surgery or other interventions. Future studies should further explore how macrophage activity and uptake processes interact with these different pathways to refine therapeutic strategies.

## 5 Limitation

The limitations of this study mainly concern the variations in immunostaining. This is particularly illustrated by the finding that two samples did not stain positive for CD68, but did stain positive of CD163 and CD192, and that 7 samples did stain positive for HE, but that presence of inflammatory cells could not be confirmed by subsequent CD68 staining. This is a limitation of immunostaining in general. Furthermore, the numbers of immunohistochemically stained macrophages varied widely and the number of CD163 and/or CD192 were frequently outranking the numbers of the CD68 staining. This may be explained as follows: the set of immunohistochemically stained slices (from the same patient) were adjacent slices in pathological wax block sections, resulting in not to fully encompass the same cells. However, after all images were evaluated, 85 % were assessed as highly comparable, ensuring a certain degree of reliability in the data. As a result, data distribution in this study is non-normal and the variance is unequal, making the ‘inflammation’ variables less suitable to analyze as a numerical variable and more suitable as a categorical variable instead. Unfortunately, this made us unable to properly correct for confounding factors in statistical analysis. At last, since we did not use fat suppression MRI sequences in our study, it is possible that MC type I may have been overlooked and that some of the MC type II patients also display MC type I, which may explain why M1 macrophage dominance is still observed occasionally in the MC type II patients.

## 6 Conclusion

It was demonstrated that Modic type I changes are associated with severe M1 macrophage-dominant inflammation. This fits very well to the findings of antibiotics being more successful in patients with MC type I changes. Moreover, it was demonstrated that both larger and non-contained herniated nucleus pulposus sizes associated with an increased number of inflammatory cells, and presence of MC type II. This may suggest that mechanical compression is a more dominant factor in radiculopathy in those patients demonstrating MC type II presence on the herniated disc level. These findings bring us one step nearer developing personalized treatment strategies in patients suffering from LRS.

## Declaration of competing interest

This work was supported by China Scholarship Council (CSC) and the Department of Neurosurgery, Leiden University Medical Center (LUMC), The Netherlands. Wensen Li received support from both funding agencies. The CSC and LUMC did not play a role in the design of the study, data collection or analysis of the data. For the remaining authors none were declared.
